# Supplementary material for: Flood disasters and health among the urban poor
Source: Health Econ. 2022 Jun 30;31(9):2072–89. doi: 10.1002/hec.4566 (PMC9546021; doi:10.1002/hec.4566)
Supplement: Supplementary file 1 — Supporting Information S1 [file HEC-31-2072-s001.docx]

# **Appendix**

**Table A1**. Descriptive statistics of IFLS respondents that are poor in urban areas of Indonesia

|  | Adult Sample | |  | Child Sample | |
| --- | --- | --- | --- | --- | --- |
| Variables | IFLS4 | IFLS5 |  | IFLS4 | IFLS5 |
|  | (1) | (2) |  | (3) | (4) |
| **Demographic Characteristics** |  |  |  |  |  |
| Age now | 39.66 | 46.50 |  | 6.66 | 13.76 |
| Gender: Male | 0.46 | 0.45 |  | 0.51 | 0.49 |
| Marital Status - Unmarried / Head (children) | 0.24 | 0.12 |  | - | - |
| Marital Status - Married / Head (children) | 0.62 | 0.69 |  | 0.84 | 0.77 |
| Marital Status - Separated / Head (children) | 0.01 | 0.01 |  | 0.03 | 0.03 |
| Marital Status - Divorced / Head (children) | 0.03 | 0.04 |  | 0.12 | 0.11 |
| Marital Status - Widow / Head (children) | 0.10 | 0.14 |  | 0.02 | 0.08 |
| Household member has 6th grade or < | 0.48 | 0.48 |  | 0.81 | 0.40 |
| Still in School | - | - |  | 0.50 | 0.73 |
| Main activity: Working | 0.56 | 0.58 |  | 0.01 | 0.15 |
| Main activity: Looking for work | 0.01 | 0.01 |  | 0.00 | 0.01 |
| Main activity: Student | 0.06 | 0.00 |  | 0.81 | 0.71 |
| Main activity: Housekeeper | 0.23 | 0.27 |  | 0.00 | 0.05 |
| Main activity: Retired | 0.05 | 0.05 |  | 0.00 | 0.00 |
| Main activity: Unemployed | 0.08 | 0.06 |  | 0.18 | 0.07 |
| Main activity: Sick | 0.01 | 0.03 |  | 0.00 | 0.01 |
| Relation to head: Children (biological) | 0.24 | 0.15 |  | 0.68 | 0.67 |
| Relation to head: Children (step/adopted) | 0.01 | 0.01 |  | 0.02 | 0.03 |
| Relation to head: Grandchild | 0.02 | 0.01 |  | 0.27 | 0.13 |
| Relation to head: Nephews/nieces | 0.01 | 0.00 |  | 0.02 | 0.03 |
| Relation to head: Other | 0.72 | 0.84 |  | 0.01 | 0.13 |
| Age of household head | 50.96 | 51.39 |  | 45.32 | 45.31 |
| Household head has 6th grade or < | 0.45 | 0.38 |  | 0.39 | 0.39 |
| Activity of head: Working | 0.72 | 0.71 |  | 0.78 | 0.75 |
| Activity of head: Housekeeper | 0.09 | 0.11 |  | 0.09 | 0.10 |
| Activity of head: Retired | 0.09 | 0.07 |  | 0.04 | 0.03 |
| Activity of head: Unemployed | 0.08 | 0.07 |  | 0.06 | 0.06 |
| Activity of head: Sick | 0.02 | 0.03 |  | 0.01 | 0.02 |
| Expenditure quintile: Poorest | 0.44 | 0.53 |  | 0.43 | 0.48 |
| Expenditure quintile: Poorer | 0.41 | 0.22 |  | 0.44 | 0.23 |
| Expenditure quintile: Middle | 0.08 | 0.12 |  | 0.06 | 0.13 |
| Expenditure quintile: Richer | 0.05 | 0.08 |  | 0.05 | 0.09 |
| Expenditure quintile: Richest | 0.02 | 0.05 |  | 0.02 | 0.06 |
| Material used in walls is non-porous | 0.79 | 0.85 |  | 0.77 | 0.84 |
| Floor is made of dirt or unfinished | 0.05 | 0.03 |  | 0.05 | 0.03 |
| Number of children < 14 per household | 1.28 | 1.17 |  | 2.32 | 1.59 |
| Number of members per household | 4.65 | 4.34 |  | 5.58 | 4.95 |
| **Outcome Variables** |  |  |  |  |  |
| Self-reported unhealthy | 0.16 | 0.28 |  | 0.11 | 0.11 |
| Reported at least 1 Morbidity in past 4 weeks | 0.72 | 0.81 |  | 0.67 | 0.80 |
| Number of Morbidities in past 4 weeks | 1.93 | 2.51 |  | 1.98 | 2.40 |
| Cough in the past 4 weeks | 0.32 | 0.42 |  | 0.38 | 0.43 |
| Difficulty breathing in the past 4 weeks | 0.07 | 0.09 |  | 0.03 | 0.05 |
| Stomach ache in the past 4 weeks | 0.19 | 0.29 |  | 0.17 | 0.34 |
| Nausea in the past 4 weeks | 0.10 | 0.16 |  | 0.10 | 0.13 |
| Diarrhea in the past 4 weeks | 0.07 | 0.11 |  | 0.11 | 0.09 |
| Skin infection in the past 4 weeks | 0.09 | 0.15 |  | 0.07 | 0.12 |
| CES-D 10 Scale | 6.26 | 8.51 |  | - | - |
| Individual is depressed | 0.13 | 0.36 |  | - | - |
| Observations | 4878 | 3950 |  | 1997 | 2379 |
| **Note.** - Mean statistics reported | | | | | |

**Table A2.** Descriptive statistics for RISE adults and children at baseline in informal settlements of Makassar, Indonesia

| Variables | Adult Sample | Child Sample |
| --- | --- | --- |
|  | (1) | (2) |
| **Demographic Characteristics** |  |  |
| Age | 41.47 | 6.51 |
| Gender: Male | 0.10 | 0.53 |
| Number of people in household | 4.78 | 5.57 |
| Children under 15 | 1.42 | 2.21 |
| Lived <6 months in settlement | 0.01 | 0.01 |
| Lived 6 months-1 year | 0.02 | 0.01 |
| Lived 1-2 years in settlement | 0.04 | 0.04 |
| Lived 2-5 years in settlement | 0.09 | 0.11 |
| Lived 5-10 years in settlement | 0.12 | 0.16 |
| Lived >10 years in settlement | 0.44 | 0.41 |
| Lived all life in settlement | 0.28 | 0.25 |
| Freehold Ownership Title | 0.50 | 0.49 |
| Sale & Purchase Deed | 0.27 | 0.27 |
| Temporary Registration Letter | 0.11 | 0.12 |
| Right to Work Land | 0.01 | 0.02 |
| Proof of Payment/Instalment | 0.03 | 0.04 |
| Relocation Letter | 0.02 | 0.01 |
| Tenure - Other | 0.05 | 0.04 |
| Roofing - Corrugated tin / iron / alum / zinc | 1.00 | 1.00 |
| Material used in walls is porous | 0.53 | 0.48 |
| Walls - Masonry | 0.72 | 0.75 |
| Walls - Wood or plywood | 0.30 | 0.25 |
| Walls - Bamboo woven or mat | 0.02 | 0.02 |
| Walls - Tin or corrugated iron | 0.39 | 0.35 |
| Flood is made of dirt or unfinished | 0.42 | 0.37 |
| Flooring - Ceramic / tiles / terrazzo | 0.57 | 0.59 |
| Flooring - Laminate (plastic) | 0.10 | 0.09 |
| Flooring - Concrete | 0.46 | 0.48 |
| Flooring - Wood/boards | 0.41 | 0.36 |
| Flooring - Bamboo | 0.01 | 0.01 |
| Flooring - Soil/dirt | 0.03 | 0.03 |
| Open Sewage | 0.10 | 0.09 |
| Unprotected Water | 0.54 | 0.56 |
| In past 3 months flood outside/under house | 0.11 | 0.12 |
| **Outcome Variables** |  |  |
| Poor health of respondent/child | 0.39 | 0.22 |
| In last month, how many days where you sick to do normal act. | 1.53 | - |
| Respondent had at least 1 acute morbidity | 0.42 | 0.44 |
| Respondent number of morbidities | 0.64 | 0.69 |
| In last 3 months, have you seen health worker | 0.25 | 0.33 |
| In the last week, have you had a cough lasting through day? | 0.16 | 0.19 |
| In the last week, have you had trouble breathing? | 0.07 | 0.02 |
| In the last week, have you had a fever? | 0.11 | 0.21 |
| In the last week, did you have three or + loose stools in 24h? | 0.06 | 0.08 |
| In the last week, how many days did you have 3+ loose stools? | 0.14 | 0.20 |
| In the last week, have you had a skin infection? | 0.12 | 0.09 |
| CESD Depression 10 Scale / PedsQL Score | 6.70 | 18.74 |
| Individual is depressed | 0.18 | - |
| Observations | 500 | 579 |

**Note. -** Mean statistics reported at baseline

| **Table A3.** Predicting the probability of attrition in IFLS5 | | | |  |
| --- | --- | --- | --- | --- |
| Variables | Attrition | Attrition:  Passed Away | Attrition:  Left sample | |
|  | (1) | (2) | (3) | |
| Flood in IFLS4 | -0.003 | -0.002 | -0.001 | |
|  | (0.006) | (0.004) | (0.005) | |
| Gender: Male | 0.022*** | 0.024*** | -0.002 | |
|  | (0.003) | (0.002) | (0.003) | |
| Age of respondent in 2007 | 0.006*** | 0.005*** | 0.001*** | |
|  | (0.000) | (0.000) | (0.000) | |
| Working in 2007 | -0.073*** | -0.058*** | -0.015*** | |
|  | (0.004) | (0.003) | (0.003) | |
| High Education | -0.006** | -0.012*** | 0.006*** | |
|  | (0.003) | (0.002) | (0.002) | |
| Relation to head: Spouse | -0.013** | -0.008** | -0.005 | |
|  | (0.005) | (0.004) | (0.004) | |
| Relation to head: Children | 0.058*** | 0.047*** | 0.011** | |
|  | (0.006) | (0.004) | (0.005) | |
| Relation to head: Other | 0.076*** | 0.071*** | 0.005 | |
|  | (0.007) | (0.006) | (0.005) | |
| Married in 2007 | -0.028*** | -0.017*** | -0.011*** | |
|  | (0.005) | (0.004) | (0.004) | |
| Log Consumption Expenditure | 0.025*** | -0.005*** | 0.030*** | |
|  | (0.003) | (0.002) | (0.002) | |
| Number of household Members | -0.009*** | -0.002** | -0.007*** | |
|  | (0.001) | (0.001) | (0.001) | |
| Number of children in household | 0.010*** | 0.003** | 0.007*** | |
|  | (0.002) | (0.001) | (0.001) | |
| Province FE | YES | YES | YES | |
| Mean Attrition | 9.31% | 4.38% | 4.93% | |
| R-squared | 0.129 | 0.166 | 0.038 | |
| Observations | 38831 | 38831 | 38831 | |
| **Note. -** Model 1 controls for all covariates included in the main regression results plus province fixed effects and indicators of socio-economic status such as working status, level of education and log of consumption expenditure. Models 2 and 3 perform a similar analysis but disaggregating the type of attrition by death and leaving the sample or unable to track. Reference levels are relation to head (Head of Household), Marital Status (Single, Divorced, Widowed) High Education (Less than 1 year of secondary education). Robust standard errors in (parentheses).  * p<0.1; ** p<0.05; *** p<0.01 | | | |  |

| **Table A4.** IFLS Results for IFLS urban poor adults and children in Indonesia  with added (potentially endogenous) controls | | | | | | |
| --- | --- | --- | --- | --- | --- | --- |
|  | Adults | | |  | Children | |
| Variables | Poor Health | Number of Morbidities | CESD Score |  | Poor Health | Number of Morbidities |
|  | (1) | (2) | (3) |  | (4) | (5) |
| (A) Flood in past 5 years | 0.034 | 0.146* | 0.641** |  | 0.015 | 0.382*** |
|  | (0.024) | (0.080) | (0.290) |  | (0.032) | (0.140) |
| (B) Flood 0-1 Year Ago | 0.081* | 0.329** | 1.475*** |  | 0.006 | 0.410 |
|  | (0.042) | (0.147) | (0.502) |  | (0.060) | (0.255) |
| Flood > 1 Year Ago | 0.024 | 0.108 | 0.467 |  | 0.017 | 0.373** |
|  | (0.027) | (0.086) | (0.318) |  | (0.035) | (0.150) |
| Mean Outcome | 21.60% | 2.26 | 7.8 |  | 11.22% | 2.22 |
| Observations | 8004 | 8002 | 7590 |  | 4048 | 4045 |
| **Note.** - For the adult sample in columns 1-3, the additional controls include marital status, equivalized consumption, education level, and main activity of the household member. For the child sample in columns 4-5 we instead control for marital status, education level and primary activity of the child's caregiver and household wealth index. Robust standard errors in (parentheses).  * p<0.1; ** p<0.05; *** p<0.01 | | | | | | |

| **Table A5.** Estimated effects of floods for urban poor IFLS (Regency Fixed Effects) | | | | | | |
| --- | --- | --- | --- | --- | --- | --- |
|  |  | Adults |  |  | Children | |
| Variables | Poor Health | Acute Morbidities | Depression Score |  | Poor Health | Acute Morbidities |
|  | (1) | (2) | (3) |  | (4) | (5) |
| (A) Flood in last 5 years | 0.023 | 0.083 | 0.490* |  | 0.018 | 0.282** |
|  | (0.024) | (0.081) | (0.296) |  | (0.031) | (0.134) |
| (B) Flood 0-1 year ago | 0.058 | 0.318** | 1.312** |  | 0.022 | 0.289 |
|  | (0.039) | (0.147) | (0.517) |  | (0.058) | (0.256) |
| Flood > 1 year ago | 0.015 | 0.037 | 0.325 |  | 0.017 | 0.279* |
|  | (0.027) | (0.087) | (0.323) |  | (0.033) | (0.143) |
| Mean Outcome | 21.60% | 2.26 | 7.8 |  | 11.22% | 2.22 |
| Observations | 8006 | 8004 | 7591 |  | 4072 | 4072 |
| **Note.** - All regressions control for individual fixed effects, wave dummies, individual and household characteristics, and regency fixed effects. Individual characteristics include a cubic function of age. Household characteristics include age of household head, age of head squared, number of children under 14 in the household, number of members per household. Individuals were considered poor if their average daily equivalized income in IFLS4 and IFLS5 was below the $1.51 ADB poverty line for Asia Pacific countries. All models use data from waves 4 and 5 of the IFLS. Robust standard errors in (parentheses).  * p < 0.1; ** p < 0.05; *** p < 0.01. | | | | | | |

| **Table A6.** Checking for Endogeneity of Floods among  the IFLS Adult Sample | |
| --- | --- |
| Variables | Flood |
|  | (1) |
|  |  |
| Log Consumption Expenditure | -0.001 |
|  | (0.009) |
| Poor Health | 0.009 |
|  | (0.015) |
| Number of Morbidities | 0.000 |
|  | (0.004) |
| Marital Status: Married | 0.012 |
|  | (0.028) |
| Marital Status: Separated | -0.019 |
|  | (0.064) |
| Marital Status: Divorced | -0.025 |
|  | (0.050) |
| Marital Status: Widow | 0.005 |
|  | (0.044) |
| Activity: Looking for work | 0.027 |
|  | (0.047) |
| Activity: Student | -0.014 |
|  | (0.023) |
| Activity: Housekeeper | 0.028* |
|  | (0.016) |
| Activity: Retired | 0.002 |
|  | (0.023) |
| Activity: Unemployment | -0.032 |
|  | (0.021) |
| Activity: Sick | -0.021 |
|  | (0.051) |
| Activity: Other | -0.051 |
|  | (0.067) |
| High Education | 0.008 |
|  | (0.020) |
| Age | 0.001 |
|  | (0.003) |
| Number of household Members | -0.001 |
|  | (0.003) |
| Number of children in household | 0.000 |
|  | (0.007) |
| Province-Wave FE | YES |
| Observations | 7064 |
| R-squared | 0.042 |
| F-Test | 0.61 |
| p-value | 0.893 |
| **Note**. – To check for endogeneity of floods among the IFLS sample we regress floods in IFLS5 on health status, individual and household characteristics in IFLS4. Reported F-statistic tests for joint significance include all lags in table A6, except province-wave interactions. Robust standard errors in (parentheses).  * p<0.1; ** p<0.05; *** p<0.01. | |

**Table A7.** Estimated effects of floods on morbidities for poor IFLS respondents

in urban areas of Indonesia

| Variables | Runny Nose | Cough | Stomach Ache | Nausea | Diarrhea | Skin Infections |
| --- | --- | --- | --- | --- | --- | --- |
|  | (1) | (2) | (3) | (4) | (5) | (6) |
| (A) Adults |  |  |  |  |  |  |
| Flood in the last 5 years | 0.048 | 0.028 | 0.026 | 0.015 | 0.016 | 0.000 |
|  | (0.031) | (0.028) | (0.027) | (0.021) | (0.018) | (0.020) |
|  |  |  |  |  |  |  |
| Flood 0-1 year ago | 0.058 | 0.078 | 0.074 | 0.078** | 0.017 | 0.026 |
|  | (0.064) | (0.056) | (0.053) | (0.036) | (0.037) | (0.043) |
| Flood > 1 year ago | 0.045 | 0.018 | 0.016 | 0.002 | 0.015 | -0.005 |
|  | (0.033) | (0.029) | (0.029) | (0.022) | (0.019) | (0.022) |
| Observations | 8004 | 8004 | 8004 | 8004 | 8004 | 8004 |
|  |  |  |  |  |  |  |
| (B) Children |  |  |  |  |  |  |
| Flood in the last 5 years | 0.079* | 0.120** | 0.025 | 0.000 | 0.067*** | 0.035 |
|  | (0.047) | (0.049) | (0.041) | (0.030) | (0.026) | (0.028) |
|  |  |  |  |  |  |  |
| Flood 0-1 year ago | 0.073 | 0.205*** | -0.034 | 0.000 | 0.073 | 0.005 |
|  | (0.082) | (0.076) | (0.082) | (0.056) | (0.054) | (0.060) |
| Flood > 1 year ago | 0.079 | 0.097* | 0.041 | 0.000 | 0.065** | 0.043 |
|  | (0.051) | (0.054) | (0.042) | (0.032) | (0.029) | (0.031) |
|  |  |  |  |  |  |  |
| Observations | 4070 | 4070 | 4070 | 4070 | 4070 | 4070 |
| **Note.** - Individual characteristics controlled for include a cubic function of age. Household characteristics controlled for include age of household head, age of head squared, number of children under 14 in the household, number of members per household. All morbidities reportedly occurred in the 4 weeks prior to the interview. All models use data from waves 4 and 5 of the IFLS. Robust errors in (parentheses).  * p < 0.1; ** p < 0.05; *** p < 0.01. | | | | | | |

| **Table A8.** Estimated effects of floods by quintiles of IFLS urban consumption expenditure (Top 3 quintiles) | | | | | | |
| --- | --- | --- | --- | --- | --- | --- |
|  |  | Adults |  |  | Children | |
| Variables | Poor Health | Acute Morbidities | Depression Score |  | Poor Health | Acute Morbidities |
|  | (1) | (2) | (3) |  | (4) | (5) |
| (A) Quintile 3 |  |  |  |  |  |  |
| Flood in last 5 years | 0.048 | -0.156 | 0.455 |  | 0.046 | -0.022 |
|  | (0.032) | (0.099) | (0.339) |  | (0.034) | (0.145) |
| Flood 0-1 year ago | 0.039 | -0.281 | 0.353 |  | 0.008 | 0.233 |
|  | (0.057) | (0.193) | (0.594) |  | (0.077) | (0.252) |
| Flood > 1 year ago | 0.050 | -0.127 | 0.480 |  | 0.053 | -0.078 |
|  | (0.036) | (0.108) | (0.379) |  | (0.035) | (0.158) |
| (B) Quintile 4 |  |  |  |  |  |  |
| Flood in last 5 years | 0.012 | 0.092 | 0.079 |  | 0.048 | 0.148 |
|  | (0.025) | (0.082) | (0.270) |  | (0.031) | (0.116) |
| Flood 0-1 year ago | 0.026 | 0.368** | 0.635 |  | -0.079 | 0.365 |
|  | (0.042) | (0.171) | (0.502) |  | (0.064) | (0.295) |
| Flood > 1 year ago | 0.010 | 0.050 | -0.013 |  | 0.069** | 0.113 |
|  | (0.027) | (0.085) | (0.289) |  | (0.034) | (0.127) |
| (C) Quintile 5 |  |  |  |  |  |  |
| Flood in last 5 years | 0.015 | 0.021 | 0.339 |  | 0.027 | 0.034 |
|  | (0.026) | (0.083) | (0.266) |  | (0.034) | (0.118) |
| Flood 0-1 year ago | 0.030 | 0.310* | 0.781 |  | 0.039 | -0.052 |
|  | (0.053) | (0.175) | (0.519) |  | (0.066) | (0.307) |
| Flood > 1 year ago | 0.012 | -0.027 | 0.264 |  | 0.025 | 0.047 |
|  | (0.027) | (0.086) | (0.282) |  | (0.035) | (0.125) |
| **Note.** - All regressions control for individual fixed effects, wave dummies, individual and household characteristics, and province*wave fixed effects. Individual characteristics include a cubic function of age. Household characteristics include age of household head, age of head squared, number of children under 14 in the household, number of members per household. All models use data from waves 4 and 5 of the IFLS. Robust standard errors in (parentheses).  * p < 0.1; ** p < 0.05; *** p < 0.01. | | | | | | |

| **Table A9.** Estimated effects of floods on health on the IFLS urban sample using different poverty definitions | | | | | | | | |
| --- | --- | --- | --- | --- | --- | --- | --- | --- |
|  | Adults | | | | |  | Children | |
| Variables | Acute Morbidities | |  | Depression Score | |  | Acute Morbidities | |
|  | Low Prosperity | Raskin 30% |  | Low Prosperity | Raskin 30% |  | Low Prosperity | Raskin 30% |
|  | (1) | (2) |  | (3) | (4) |  | (5) | (6) |
| (A) Flood in last 5 years | 0.039 | 0.008 |  | 0.452* | 0.384 |  | 0.258** | 0.236** |
|  | (0.069) | (0.094) |  | (0.264) | (0.240) |  | (0.103) | (0.107) |
| (B) Flood 0-1 year ago | 0.177 | 0.282 |  | 0.820* | 1.403*** |  | 0.539** | 0.622*** |
|  | (0.143) | (0.191) |  | (0.493) | (0.417) |  | (0.218) | (0.235) |
| Flood > 1 year ago | 0.020 | -0.034 |  | 0.400 | 0.084 |  | 0.206* | 0.141 |
|  | (0.072) | (0.100) |  | (0.275) | (0.278) |  | (0.111) | (0.124) |
| Mean Outcome | 1.43 | 1.40 |  | 8.08 | 8.09 |  | 1.48 | 1.47 |
| Observations | 11448 | 7985 |  | 10954 | 10981 |  | 5087 | 5096 |
| **Note.** – Columns 1, 3, 5 (Low Prosperity) restrict the sample to the panel of adults and children living in an IFLS village whose head identified as being in the bottom three steps of a six-step prosperity ladder in IFLS4. Columns 2, 4, 6 (Raskin 30%) restricts the sample to the panel of individuals living in urban villages where at least 30% of the households received subsidies for the purchase of rice in IFLS4 as part of the Raskin program targeted to the poor. All regressions control for individual fixed effects, wave dummies, individual and household characteristics, and province*wave fixed effects. All models use data from waves 4 and 5 of the IFLS. Robust standard errors in (parentheses).  * p < 0.1; ** p < 0.05; *** p < 0.01. | | | | | | | | |

| **Table A10.** Correlation between PODES floods and reporting of floods in IFLS households | |
| --- | --- |
| Variables | (1) |
|  | HHD Flood |
|  |  |
| Age | -0.006 |
|  | (0.006) |
| At least 1 year of high school | -0.029 |
|  | (0.020) |
| Poor Health | -0.003 |
|  | (0.011) |
| CESD | 0.002* |
|  | (0.001) |
| Percentage of villages flooded in district | 0.198* |
|  | (0.118) |
| Percentage of villages flooded in district * Age | 0.001 |
|  | (0.002) |
| Percentage of villages flooded in district * Female | 0.072 |
|  | (0.059) |
| Percentage of villages flooded in district * Secondary Education | 0.082 |
|  | (0.062) |
| Percentage of villages flooded in district * Poor health | 0.050 |
|  | (0.042) |
| Percentage of villages flooded in district * CESD | 0.000 |
|  | (0.004) |
| F-test (interactions) | 0.90 |
| p-value (interactions) | 0.4806 |
| Individual FE | Yes |
| Prov*Year FE | Yes |
| Mean Outcome | 11.64% |
| Observations | 33979 |
| **Note. –** The variable percentage of villages flooded was rescaled from 0-100 to a 0-10 scale. All models use data from waves 4 and 5 of the IFLS. Robust standard errors in (parentheses).  * p < 0.1; ** p < 0.05; *** p < 0.01. | |

| **Table A11.** Checking for flood endogeneity among the RISE sample | | | |
| --- | --- | --- | --- |
| Variables | Any Damage from Floods | | |
|  | Adults |  | Children |
|  | (1) |  | (2) |
| Poor health | -0.014 |  | -0.047 |
|  | (0.040) |  | (0.043) |
| Number of morbidities | -0.007 |  | -0.008 |
|  | (0.021) |  | (0.020) |
| CESD-10 Score | -0.004 |  | - |
|  | (0.005) |  |  |
| Poorer Quintile | -0.013 |  | 0.011 |
|  | (0.054) |  | (0.056) |
| Middle Quintile | -0.042 |  | -0.015 |
|  | (0.062) |  | (0.059) |
| Richer Quintile | -0.037 |  | -0.031 |
|  | (0.056) |  | (0.049) |
| Richest Quintile | -0.092 |  | -0.062 |
|  | (0.063) |  | (0.053) |
| Age | -0.002 |  | 0.000 |
|  | (0.002) |  | (0.004) |
| Gender: Male | 0.073 |  | -0.034 |
|  | (0.063) |  | (0.033) |
| Number of children in household | -0.029 |  | 0.025 |
|  | (0.023) |  | (0.023) |
| Number of people in household | 0.010 |  | -0.011 |
|  | (0.013) |  | (0.012) |
| Marital Status: Married | 0.105 |  | 0.156* |
|  | (0.078) |  | (0.093) |
| Marital Status: Other | 0.133 |  | 0.225* |
|  | (0.097) |  | (0.122) |
| Primary Activity: Employed | -0.011 |  | -0.015 |
|  | (0.046) |  | (0.044) |
| Has Secondary Schooling or Higher | 0.082** |  | 0.069* |
|  | (0.040) |  | (0.035) |
| Settlement FE | YES |  | YES |
| Observations | 489 |  | 579 |
| R-squared | 0.338 |  | 0.301 |
| F-test | 0.83 |  | 0.94 |
| p-value | 0.643 |  | 0.5119 |
| **Note.** – All independent variables were measured at baseline prior to the flood. For the child sample in column 2, the variables marital status, primary activity and level of education correspond to their caregivers. The reference levels are Quintile (Poorest), Gender (Female), Marital Status (Single), Main Activity (Unemployed, seeking job or housewife), Schooling (Less than one year of secondary schooling or no schooling at all). F-test for all controls except settlement FE. Robust standard errors in (parentheses).  * p<0.1; ** p<0.05; *** p<0.01. | | | |

**Table A12.** RISE Alternative Specification (Settlement Fixed Effects)

| Variables | Poor Health | Acute Morbidities | Depression Score /  Emotional Problems |
| --- | --- | --- | --- |
|  | (1) | (2) | (3) |
| (A) Adults |  |  |  |
| 4-5 months after | 0.136** | 0.318* | - |
|  | (0.058) | (0.150) |  |
| 10-11 months after | 0.030 | 0.020 | 1.302* |
|  | (0.050) | (0.150) | (0.660) |
| Mean Outcome | 28% | 0.53 | 6.49 |
| Observations | 1429 | 1429 | 897 |
| (B) Children |  |  |  |
| 4-5 months after | 0.109 | 0.195* | 12.325*** |
|  | (0.117) | (0.095) | (2.063) |
| 10-11 months after | -0.019 | -0.064 | 5.864** |
|  | (0.049) | (0.082) | (2.006) |
| Mean Outcome | 17% | 0.59 | 17.38 |
| Observations | 1716 | 1715 | 1030 |
| **Note. -** All models control for individual and household characteristics as well as wave fixed effects and settlement*wave interactions. Individual characteristics include: age and gender. Household characteristics include: number of children and number of people in the household. House characteristics include: a list of all assets in the house at baseline, material of floor, roof and walls. All models include data from three waves: Baseline, Wave 2 (4-5 months post flood) and Wave 3 (10-11 months post flood). Robust standard errors in (parentheses).  * p<0.1; ** p<0.05; *** p<0.01. | | | |
